# On the Total Synthesis of 7,8(*S*,*S*)-Epoxy-17(*S*)-hydroxy-4(*Z*),9(*E*),11(*E*),13(*Z*),15(*E*),19(*Z*)-docosahexaenoic Acid Derivative

**DOI:** 10.3390/molecules30081858

**Published:** 2025-04-21

**Authors:** Robert Nshimiyimana, Charles N. Serhan, Nicos A. Petasis

**Affiliations:** 1 Center for Experimental Therapeutics and Reperfusion Injury, Department of Anesthesiology, Perioperative and Pain Medicine, Brigham and Women’s Hospital and Harvard Medical School, Boston, MA 02115, USA; 2 Department of Chemistry and Loker Hydrocarbon Research Institute, University of Southern California, Los Angeles, CA 90089, USA

**Keywords:** stereoselective total synthesis, specialized pro-resolving mediators, resolvins, natural products

## Abstract

The stereoselective total synthesis of an allylic epoxide-containing polyunsaturated fatty acid, in its triethylsilyl (TES) ether and methyl ester form, is described. Key features include a Sharpless enantioselective epoxidation to install the oxirane unit and Wittig coupling reactions to forge critical alkenyl configuration and secure the core carbon skeleton. The deprotected epoxy acid was recently demonstrated to play a central role as the precursor to biologically active resolvins D1, D2, and the cysteinyl conjugate in tissue regeneration (RCTR1) by human leukocytes. These natural products belong to a family of cell signaling molecules termed specialized pro-resolving mediators (SPMs).

## 1. Introduction

In 1971, a landmark discovery on blood lipid composition in native Greenland Inuit by Bang and Dyerberg lit the fuse of ω-3 fatty acid research [1]. Their pioneering observations led to formulation of a hypothesis to account for the low incidence of myocardial infarction in a population consuming a high-fat and high-cholesterol diet [1,2]. This work threw open the floodgates of investigations and stimulated interest in ω-3 polyunsaturated fatty acids (PUFAs), primarily docosahexaenoic acid (DHA) and eicosapentaenoic acid (EPA).

First reported by Serhan and coworkers at the turn of the 21st century [3], evidence that enzymatic products from ω-3 essential fatty acids are potent regulators and stop signals of inflammation has increased ever since, as witnessed by the daily influx of literature describing their total synthesis, biosynthesis, biological activities and pharmacology in this rapidly expanding field [4,5,6,7,8,9,10,11]. These versatile, biologically active molecules are formally known as specialized pro-resolving mediators (SPMs) [12], which include the resolvins, protectins, maresins, and lipoxins. The elucidation of each structure has resulted from multidisciplinary efforts combining the identification and isolation of active products in humans and other natural sources, total synthesis, and spectroscopic studies, to name but a few.

**Figure 1:**
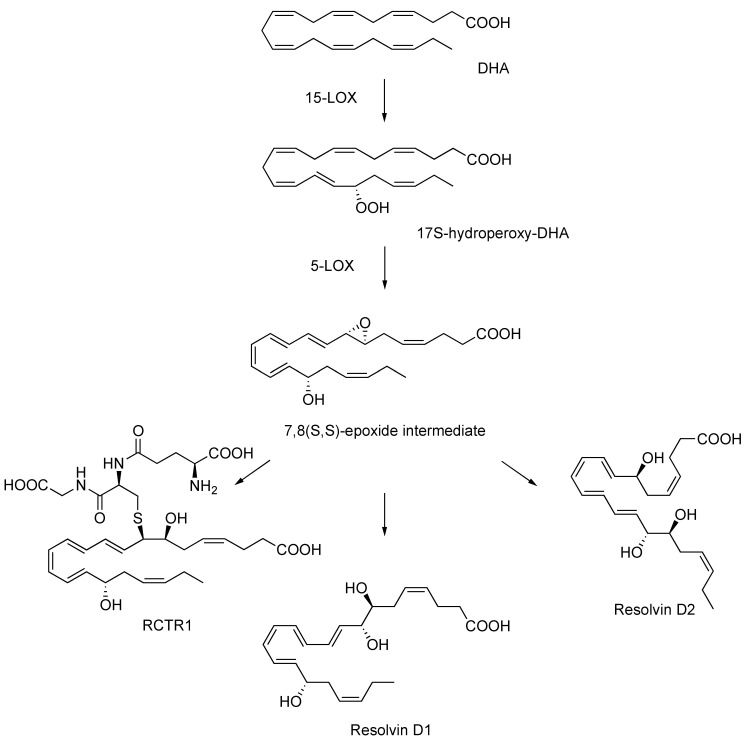
Proposed biosynthesis of resolvin D1, resolvin D2, and RCTR1 via the 7,8(*S*,*S*)-epoxide intermediate.

In this report, we detail the total synthesis towards 7,8(*S*,*S*)-epoxy-17(*S*)-hydroxy-4(*Z*),9(*E*),11(*E*),13(*Z*),15(*E*),19(*Z*)-docosahexaenoic acid (Figure 1) [13], a key predecessor of potent anti-inflammatory and pro-resolving lipid mediators.

This transient epoxy acid was recently revealed to be a direct intermediate for the endogenous formation of resolvin D1, resolvin D2, and the sulfhydroxyl resolvin conjugate in tissue regeneration (RCTR1) by isolated human leukocytes and recombinant enzymes [13]. These studies with the allylic epoxide utilized analytical approaches, including O^18^ isotope labeling, ion trap mass spectrometry techniques, and in silico molecular docking simulations, thus providing definitive evidence of its participation in the biosynthesis of the potent natural products [13] and adding a central piece to the puzzle of resolution mechanisms, one molecule at a time.

The proposed biosynthetic transformation of DHA to resolvin D1, resolvin D2, and RCTR1 involves sequential oxygenation by 15-lipoxygenase (15-LOX) to generate 17*S*-hydroperoxy-DHA, followed by 5-LOX to produce the 7,8(*S*,*S*)-epoxy-intermediate. Further processes by specific cell types and enzymes lead to the production of these inflammation stop signals (Figure 1) [13]. Due to their important physiological roles, it is of broad interest to elucidate the endogenous mechanisms and intermediates involved in the biosynthesis of these biologically active agents.

**Figure 2:**
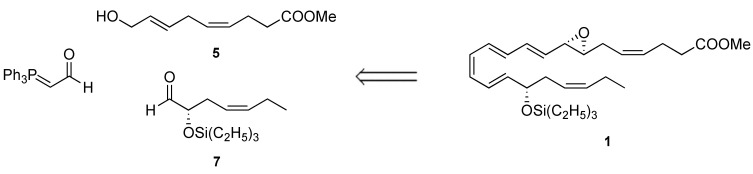
Retrosynthetic analysis of compound **1**.

Because of their potency and limited endogenous availability, resolvins require preparation by total synthesis to enable detailed biological evaluation. To date, only Rodriguez and Spur have reported the synthesis of the 7,8(*S*,*S*)-epoxy intermediate with a *tert*-butyldiphenylsilyl group and methyl ester using a chiral pool-based and enzymatic hydroxylation approach [14]. To complement these previous efforts and enable further biological examination of this labile natural product, we devised another synthetic strategy using a stereoselective and convergent approach, as illustrated in the retrograde analysis in Figure 2. 

A strategy based on chirally catalyzed oxidation reactions and Wittig olefinations was employed, with allylic alcohol **5**, *α*-siloxy aldehyde **7**, and (triphenylphosphoranylidene) ethanal identified as important pieces and linchpins for forward steps. For stability purposes, we prepared and stored the material as its triethylsilyl ether and methyl ester derivative **1**, and the protective groups were readily removed upon need for biosynthetic studies [13].

## 2. Results and Discussion

**Scheme 1:**
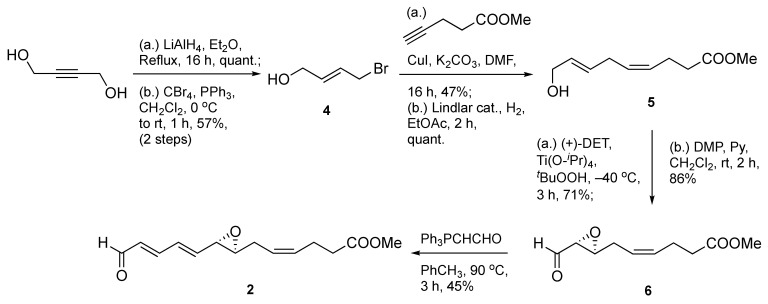
Synthesis of epoxydienal intermediate **2**.

The synthesis commenced with the preparation of the C_1_–C_13_ epoxydienal fragment **2** in a seven-step sequence from 1,4-butynediol as a non-chiral synthon (Scheme 1). Key steps included a reduction with LiAlH_4_ to create a prochiral allylic alcohol which was easily transformed as its monobromide derivative **4**. Next, a copper (I)-mediated coupling of **4** with methyl 4-pentynoate led to a 1,4-enyne-containing intermediate, followed by Lindlar reduction to afford skipped diene **5** in a quantitative manner. This allylic alcohol material was then subjected to asymmetric epoxidation under Sharpless conditions [15] to forge a 2,3(*S*,*S*)-epoxyalcohol intermediate, which was subsequently submitted to oxidation using Dess–Martin periodinane (DMP) to obtain epoxyaldehyde **6** in moderate to high yields.

Finally, the target four-carbon vinylogue (**2**) was attained in acceptable 45% yield using a homologation reaction with the stabilized Wittig reagent (triphenylphosphoranylidene)ethanal, (Ph_3_PCHCHO)) at elevated temperature. Of note, this reaction first produces a two-carbon vinylogous intermediate, which, in turn, is homologated again to the target epoxydienal.

**Scheme 2:**
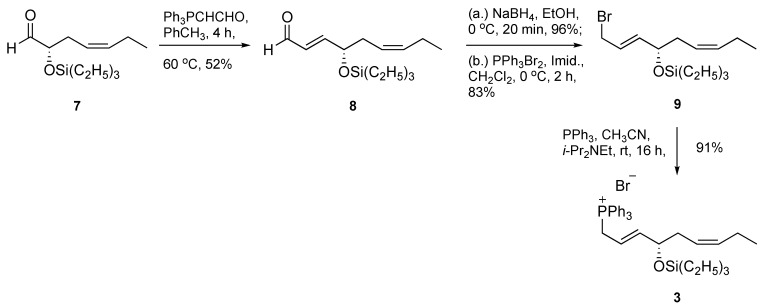
Synthesis of phosphonium bromide **3**.

Next, we set out to prepare the phosphonium salt **3** (Scheme 2). This effort began with a known and previously reported *α*-siloxy chiral aldehyde **7** [16], which was achieved using a metal-free asymmetric organocatalytic oxyamination protocol [17,18]. A two-carbon homologation yielded enal **8** in acceptable 52% yield, followed by an efficient NaBH_4_-based carbonyl reduction to furnish an allylic alcohol intermediate. Smooth conversion of the allylic alcohol to its bromide derivative **9** was accomplished under mild conditions at 0 °C using PPh_3_Br_2_ in methylene chloride in 83% yield. Next, the target C_14_–C_22_ fragment, phosphonium bromide **3**, was secured in excellent 91% yield at room temperature using triphenylphosphine in the presence of *N*,*N*-diisopropylethylamine (*i*-Pr_2_NEt). The use of *i*-Pr_2_NEt as a proton scavenger was to prevent formation of trace amounts of HBr, thus averting silyl group removal and/or potential isomerization.

**Scheme 3:**
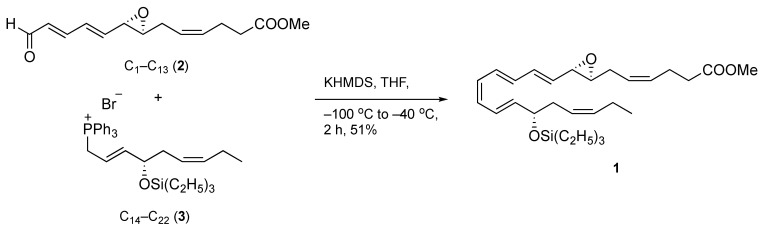
Convergent synthesis of target product **1**.

With both requisite coupling components readily available, the combination of epoxydienal **2** and phosphonium bromide **3** was achieved using a *Z*-selective Wittig olefination to assemble the carbon backbone of the target structure (Scheme 3). We expected the *Z*-stereoselective coupling of **2** and **3** to pose a challenge since our experience, on the synthesis of a related epoxy acid, and that of others, has shown that this transformation tends to favor *E*-olefination [14,16,19]. Thus, the phosphorane of **3** was prepared in situ with potassium hexamethyldisilazide in THF at −100 °C and then allowed to react with **2** to give the desired coupling product **1**. At this juncture, the use of warmer temperatures to prepare the ylide led to lower or no yields of the olefination product.

Without workup, the reaction mixture was subjected to rapid chromatography on silica gel deactivated with triethylamine, followed by HPLC separation under straight-phase conditions to deliver pure compound **1** in 51% yield. Of merit, this step generated a 13-*trans*-isomer of **1** as a minor product (*Z*/*E* ratio 9:1), a significantly worthy and improved stereoselectivity compared to the reported 1:1 *Z*/*E* ratio [14]. The UV spectrum gave a characteristic triplet band of absorption at λ_max_ (hexane/Et_3_N, 99/1) 294, 308, 322 nm, confirming the conjugated tetraenic 7,8-oxirane chromophore (Supplementary Materials). These results were in agreement with the previously reported data [14] on the total synthesis of the *t*-butyldiphenylsilyl ether derivative of compound **1**.

**Scheme 4:**
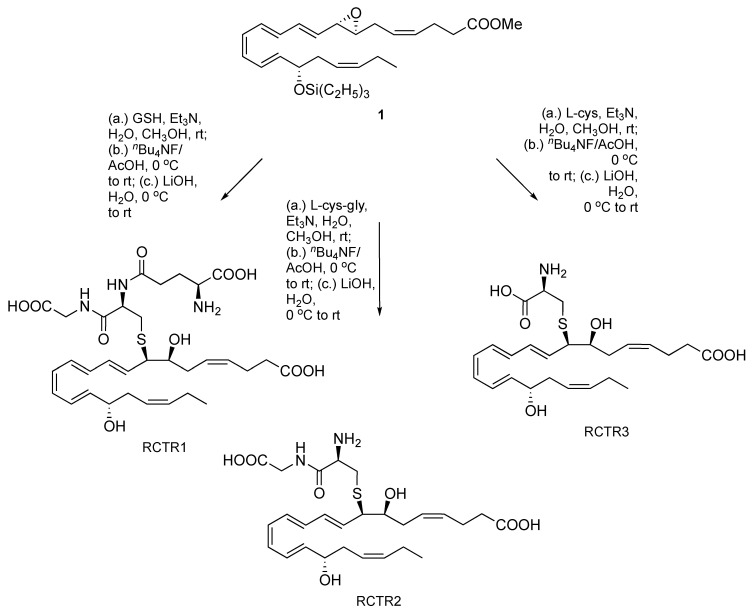
Conversion of compound **1** to RCTR1, RCTR2, and RCTR3. GSH, glutathione; L-cys-gly, L-cysteinylglycine; L-cys, L-cysteinyl.

Additionally, as illustrated in Scheme 4, we confirmed the complete intact stereochemical structure by epoxide opening with reduced glutathione, l-cysteinylglycine, and l-cysteine, followed by triethylsilyl group cleavage and methyl ester saponification to afford the known and bioactive peptidyl-conjugates RCTR1, RCTR2, and RCTR3, as demonstrated using liquid chromatography-tandem mass spectrometry (LC-MS/MS)-based analyses (Supplementary Materials). Similarly, aqueous epoxide hydrolysis of deprotected material from both the triethylsilyl [13] and *t*-butyldiphenylsilyl [14] derivatives, synthesized separately and independently, gave an identical hydrolysis product profile.

Notably, the full structural configuration and role of the synthetic epoxide (**1**), in its desilylated and free acid form (under *^n^*Bu_4_NF/AcOH then LiOH conditions), was substantially corroborated via biochemical transformations to the known and active lipid mediators resolvin D1, resolvin D2, and RCTR1 using isolated mammalian cells, recombinant enzymes, molecular docking simulations, and a broad range of mass spectrometry-based analytical approaches in our recent report [13].

## 3. Experimental Procedures

Details of the laboratory equipment and chemical compounds are found in the Supplementary Materials.

### 3.1. (E)-4-Bromobut-2-en-1-ol (***4***)

To a solution of but-2-yne-1,4-diol (8.4 g, 102.1 mmol, 1 equiv) in THF (100 mL) was added, in a dropwise manner, a suspension of LiAlH_4_ (4.63 g, 120.4 mmol, 1.2 equiv) in THF (300 mL) at 0 °C, and the resulting reaction mixture was stirred at room temperature for 16 h. Upon completion, the reaction mixture was cooled to 0 °C and was carefully hydrolyzed by using standard procedure (5 mL water + 5 mL of 3 M NaOH + 15 mL water). Then, the stirring was continued at room temperature for a further 1 h after adding anhydrous MgSO_4_. Next, the solid was filtered through a pad of celite and rinsed with CH_2_Cl_2_ and, upon concentration, to provide the *trans*-2-butene-1,4-diol (9 g, quantitative yield) as a colorless oil: ^1^H NMR (600 MHz, chloroform-*d*) δ 5.89 (ddt, *J* = 3.4, 2.7, 0.9 Hz, 2H), 4.22–4.09 (m, 4H); ^13^C NMR (151 MHz, chloroform-*d*) δ 130.7(2), 63.1(2). Subsequently, to a solution of *trans*-2-butene-1,4-diol (2.1 g, 23 mmol) in 40 mL of anhydrous CH_2_Cl_2_ at 0 °C was added triphenylphosphine (9 g, 28 mmol) and CBr_4_ (8 g, 24 mmol) and the reaction was allowed to warm up to room temperature and stirred for 1 h. It was then quenched with saturated aqueous NH_4_Cl (125 mL) and extracted with Et_2_O (3 × 125 mL). The combined extracts were dried with Na_2_SO_4_ and evaporated to give a crude brown oil, which was then chromatographed on silica gel using 10% EtOAc/hexanes as eluent to afford title monobromide **4** (1.95 g, 57%). ^1^H NMR (600 MHz, chloroform-*d*) δ 6.01–5.85 (m, 2H), 4.22–4.15 (m, 2H), 3.97 (dt, *J* = 6.6, 0.9 Hz, 2H); ^13^C NMR (151 MHz, chloroform-*d*) δ 134.3, 127.4, 62.6, 32.1.

### 3.2. Methyl (4Z,7E)-9-Hydroxynona-4,7-dienoate (***5***)

Pentynoic acid methyl ester (1.6 g, 14.3 mmol) and monobromide **4** (1.5 g, 9.9 mmol) were mixed and dissolved in 6 mL anhydrous DMF. CuI (2.8 g, 14.4 mmol), NaI (2.2 g, 14.4 mmol) and K_2_CO_3_ (2.0 g, 14.4 mmol) were sequentially added, and the solution was stirred for 15 h at room temperature. The reaction mixture was worked up with saturated NH_4_Cl aqueous solution and extracted with ether. After removing the solvent under vacuum, the mixture was purified on a silica column with 25% EtOAc/hexanes to give the acetylenic coupling product intermediate: ^1^H NMR (600 MHz, chloroform-*d*) δ 5.90 (dtt, *J* = 15.2, 5.7, 1.8 Hz, 1H), 5.68 (dtt, *J* = 15.3, 5.3, 1.5 Hz, 1H), 4.14 (ddt, *J* = 7.1, 5.7, 2.1 Hz, 2H), 3.70 (s, 3H), 2.92 (dtd, *J* = 4.0, 2.2, 0.7 Hz, 2H), 2.56–2.48 (m, 4H), 1.35 (t, *J* = 5.9 Hz, 1H). ^13^C NMR (151 MHz, chloroform-*d*) δ 172.7, 130.6, 127.1, 80.8, 77.8, 63.4, 51.9, 33.9, 21.8, 14.9. The skipped enyne was then semi-reduced using the Lindlar catalyst. This product (0.6 g, 3.3 mmol) was dissolved in 10 mL of ethyl acetate, followed by 0.1 mL of quinoline and 0.4 g of the Lindlar catalyst. The reaction mixture was purged with hydrogen and carefully stirred and monitored by the TLC (10% EtOAc/hexanes). Upon reaction completion the mixture was filtered through celite, and the solvents were removed under vacuum to afford the title skipped diene **5** (0.6 g, quantitative yield).

### 3.3. Methyl (Z)-6-((2S,3R)-3-Formyloxiran-2-yl)hex-4-enoate (***6***)

To a flame-dried 10 mL flask charged with 25 mg 4 Å molecular sieves powder was added 1 mL of CH_2_Cl_2_. The flask was cooled down to −20 °C and 0.02 mL of (+)-diethyl tartrate, 0.03 mL of Ti(O-*i*Pr)_4_, and 0.20 mL of 5 M solution (1.0 mmol) of *t*-BuOOH were added sequentially. After stirring for 1 h, 0.2 g (1.09 mmol) of allylic alcohol **5**, dissolved in 1 mL of CH_2_Cl_2_ was cannulated into the reaction mixture dropwise and the reaction stirred further for 3 h. The mixture was then filtered over a pad of celite, concentrated, and purified on a silica column with 50% EtOAc/hexanes to afford the epoxyalcohol precursor to **3** (0.155 g, 71%): ^1^H NMR (600 MHz, chloroform-*d*) δ 5.58–5.40 (m, 2H), 3.94–3.85 (m, 1H), 3.67 (s, 3H), 3.67–3.62 (m, 1H), 3.01 (td, *J* = 5.3, 2.3 Hz, 1H), 2.97 (dt, *J* = 4.2, 2.5 Hz, 1H), 2.45–2.34 (m, 6H), 1.66–1.62 (m, 1H); ^13^C NMR (151 MHz, chloroform-*d*) δ 173.6, 130.9, 124.8, 61.7, 57.9, 55.1, 51.7, 34.0, 29.3, 23.0. Subsequently, this residue was submitted to Dess–Martin periodinane (DMP) oxidation. To a flask containing 0.13 g (0.65 mmol) of the epoxyalcohol in 5 mL of CH_2_Cl_2_ was added 400 mg of NaHCO_3_ and 400 mg of DMP reagent along with a drop of water under open air. The slurry mixture was stirred for 1 h at room temperature and reaction progress was checked by TLC. After the reaction was complete, the mixture was worked up with a 1:1 NaHCO_3_/Na_2_S_2_O_3_ saturated solution and extracted with CH_2_Cl_2_ (3 × 30 mL). Extracted fractions were dried over sodium sulfate, the solids were filtered off, and the solvents removed in vacuo. The crude residue was purified on a silica column with 20% EtOAc/hexanes to obtain epoxyaldehyde **6** (0.11 g, 86%). ^1^H NMR (400 MHz, chloroform-*d*) δ 9.03 (dd, *J* = 6.2, 2.4 Hz, 1H), 5.62–5.50 (m, 1H), 5.42 (dddd, *J* = 10.1, 7.4, 5.2, 1.5 Hz, 1H), 3.68 (d, *J* = 2.3 Hz, 3H), 3.29 (td, *J* = 5.1, 2.0 Hz, 1H), 3.18 (dt, *J* = 6.2, 2.2 Hz, 1H), 2.51 (ddd, *J* = 7.1, 5.0, 1.6 Hz, 2H), 2.42–2.30 (m, 5H). ^13^C NMR (101 MHz, chloroform-*d*) δ 198.3, 173.4, 132.1, 123.4, 58.6, 56.0, 51.8, 33.8, 28.8, 23.0.

### 3.4. Methyl (Z)-6-((2S,3S)-3-((1E,3E)-5-Oxopenta-1,3-dien-1-yl)oxiran-2-yl)hex-4-enoate (***2***)

To a 10 mL pear-shaped and flame-dried flask, 0.055 mg (0.28 mmol) of aldehyde **6** and 0.2 g (0.66 mmol) of (triphenylphosphoranylidene)acetaldehyde were dissolved in 0.8 mL of toluene and refluxed at 90 °C for 2 h. Then, (triphenylphosphoranylidene)acetaldehyde (0.05 g, 0.15 mmol) was added again and stirring continued. After another 1 h under reflux, TLC (20% EtOAc/hexanes) showed reaction completion and the mixture was concentrated in vacuo and directly subjected to purification (without workup) with 10% EtOAc/hexanes to afford the desired homologated epoxyaldehyde **2** in 43%: ^1^H NMR (600 MHz, chloroform-*d*) δ 9.58 (d, *J* = 7.9 Hz, 1H), 7.08 (ddd, *J* = 15.3, 11.0, 0.8 Hz, 1H), 6.64 (dd, *J* = 15.4, 11.0 Hz, 1H), 6.17 (dd, *J* = 15.4, 7.9 Hz, 1H), 5.98 (dd, *J* = 15.3, 7.4 Hz, 1H), 5.57–5.50 (m, 1H), 5.46 (dt, *J* = 10.6, 7.3 Hz, 1H), 3.68 (s, 3H), 3.27 (dd, *J* = 7.4, 2.0 Hz, 1H), 2.96 (td, *J* = 5.2, 2.0 Hz, 1H), 2.47–2.43 (m, 2H), 2.41–2.36 (m, 4H). ^13^C NMR (151 MHz, chloroform-*d*) δ 193.7, 173.5, 150.0, 141.2, 132.4, 131.4, 131.1, 124.3, 60.7, 56.87, 51.8, 33.9, 29.7, 23.0.

### 3.5. (S,2E,6Z)-4-((Triethylsilyl)oxy)nona-2,6-dienal (***8***)

To a solution of a previously reported *α*-siloxy aldehyde building block **7** (1 g, 1 equiv, 4.183 mmol), first utilized in the synthesis of a related polyunsaturated epoxy fatty acid [16] in toluene (36 mL) was added (triphenylphosphoranylidene)acetaldehyde (2.5 g, 2 equiv, 8.365 mmol) at room temperature. The reaction mixture was brought up to 60 °C and stirred for 4 h. It was then filtered over a pad of silica with CH_2_Cl_2_. The solvent was evaporated off under vacuum and the crude product was purified by flash chromatography (2% EtOAc/hexanes) to afford title compound **8** (586 mg, 52.2%). ^1^H NMR (400 MHz, benzene-*d*_6_) δ 9.36 (d, *J* = 7.3 Hz, 1H), 6.24 (qd, *J* = 16.2, 15.6, 5.8 Hz, 2H), 5.41 (dtt, *J* = 10.3, 7.2, 1.5 Hz, 1H), 5.32–5.18 (m, 1H), 4.06 (tdd, *J* = 6.1, 4.4, 1.1 Hz, 1H), 2.21–2.02 (m, 2H), 1.86 (qd, *J* = 7.5, 1.5 Hz, 2H), 0.88 (t, *J* = 8.0 Hz, 9H), 0.83 (t, *J* = 7.5 Hz, 3H), 0.46 (q, *J* = 8.0 Hz, 6H). ^13^C NMR (101 MHz, benzene-*d*_6_) δ 191.8, 157.6, 134.2, 130.8, 123.3, 71.4, 35.2, 20.7, 13.9, 6.6(3), 4.7(3).

### 3.6. (((S,2E,6Z)-1-Bromonona-2,6-dien-4-yl)oxy)triethylsilane (***9***)

To a solution of **8** (742 mg, 1 equiv, 2.76 mmol) in wet ethanol (10 mL) was added NaBH_4_ (105 mg, 1 equiv, 2.76 mmol) at 0 °C. The reaction was stirred for 20 min. Saturated sodium bicarbonate solution was added and the product was extracted using Et_2_O, dried over MgSO_4_, filtered and concentrated under reduced pressure. The residue was then purified by column chromatography (20% EtOAc/hexanes) to yield the corresponding allylic alcohol intermediate (714 mg, 95.5%). ^1^H NMR (400 MHz, benzene-*d*_6_) δ 5.70–5.59 (m, 2H), 5.53–5.33 (m, 2H), 4.11 (tt, *J* = 6.3, 2.5 Hz, 1H), 3.79 (s, 2H), 2.37 (dt, *J* = 14.3, 6.0 Hz, 1H), 2.31–2.14 (m, 1H), 2.05–1.88 (m, 2H), 0.99 (t, *J* = 7.9 Hz, 9H), 0.88 (t, *J* = 7.4 Hz, 3H), 0.60 (q, *J* = 7.9 Hz, 6H). ^13^C NMR (101 MHz, benzene-*d*_6_) δ 133.7, 133.2, 129.2, 124.8, 72.8, 62.4, 36.4, 20.8, 14.0, 6.8(3), 5.1(3).

Subsequently, bromotriphenylphosphonium bromide (468 mg, 1.2 equiv, 1.11 mmol) was added to a flame-dried flask and the reagent was dried under high vacuum for 1 h. To a solution of the dried reagent in methylene chloride (10 mL) at 0 °C was added imidazole (94.4 mg, 1.5 equiv, 1.39 mmol) and the allylic alcohol (250 mg, 1 equiv, 924 μmol) via cannula at 0 °C. The reaction was stirred at 0 °C for 2 h until TLC indicated reaction completion, at which point it was diluted with methylene chloride and filtered on a pad of celite. The crude product was purified by column chromatography (3% EtOAc/hexanes) to yield title compound **9** (257 mg, 83.4%) ^1^H NMR (400 MHz, benzene-*d*_6_) δ 5.70 (dtd, *J* = 15.1, 7.6, 1.2 Hz, 1H), 5.56–5.38 (m, 3H), 4.07–3.99 (m, 1H), 3.49 (d, *J* = 7.5 Hz, 2H), 2.31 (dt, *J* = 13.4, 6.5 Hz, 1H), 2.21 (dt, *J* = 14.8, 6.6 Hz, 1H), 1.99 (p, *J* = 7.2 Hz, 2H), 1.02 (t, *J* = 8.0 Hz, 9H), 0.91 (t, *J* = 7.6 Hz, 3H), 0.61 (q, *J* = 7.9 Hz, 6H). ^13^C NMR (101 MHz, benzene-*d*_6_) δ 137.9, 133.5, 125.7, 124.3, 72.1, 36.0, 31.7, 20.7, 14.0, 6.8(3), 4.9(3).

### 3.7. Triphenyl((S,2E,6Z)-4-((Triethylsilyl)oxy)nona-2,6-dien-1-yl)phosphonium Bromide (***3***)

To a solution of **9** (245 mg, 1 equiv, 735 μmol) in acetonitrile (5 mL) was added DIPEA (285 mg, 0.39 mL, 3 equiv, 2.20 mmol), followed by triphenylphosphine (289 mg, 1.5 equiv, 1.10 mmol). The reaction was stirred at room temperature for 16 h. Purification was carried out by trituration with pentane until TLC showed no presence of triphenylphosphine, to afford phosphonium salt **3** (400 mg, 91.4%). ^1^H NMR (400 MHz, acetonitrile-*d*_3_) δ 8.00–7.50 (m, 15H), 5.95–5.79 (m, 1H), 5.63–5.47 (m, 1H), 5.45–5.31 (m, 1H), 5.21–5.00 (m, 1H), 4.18 (q, *J* = 5.6 Hz, 1H), 4.15–4.06 (m, 2H), 2.22–2.02 (m, 2H), 1.99–1.86 (m, 2H), 0.91 (t, *J* = 7.5 Hz, 3H), 0.84 (t, *J* = 7.9 Hz, 9H), 0.48 (qd, *J* = 7.9, 3.2 Hz, 6H). ^13^C NMR (101 MHz, acetonitrile-*d*_3_) δ 143.8 (d, *J* = 13.0 Hz), 135.2 (d, *J* = 3.0 Hz), 133.8 (d, *J* = 9.6 Hz), 130.2 (d, *J* = 12.6 Hz), 123.8, 114.0 (d, *J* = 9.5 Hz), 71.6 (d, *J* = 2.4 Hz), 35.3 (d, *J* = 3.4 Hz), 26.4 (d, *J* = 51.0 Hz), 20.4, 13.5, 6.2(3), 4.4(3).

### 3.8. Methyl (Z)-6-((2S,3S)-3-((S,1E,3E,5Z,7E,11Z)-9-((Triethylsilyl)oxy)tetradeca-1,3,5,7,11-pentaen-1-yl)oxiran-2-yl)hex-4-enoate (***1***)

The phosphonium bromide **3** (86 mg, 0.144 mmol) was dried under high vacuum and P_2_O_5_ in a reaction flask for 15 h. Then 1 mL of still-dried THF was added and the mixture was cooled to −100 °C. To the solution, a 1 M KHMDS solution in THF (0.137 mmol, 0.95 equiv) was added via cannula and the mixture was stirred at −100 °C for 1 min, followed by the addition of epoxydienal **2** (15 mg, 0.06 mmol, 0.4 equiv). The reaction was then warmed up to −40 °C and gently stirred for 2 h. The reaction material was then subjected to rapid chromatography on silica gel deactivated with triethylamine, without workup or removal of solvent, using a Et_3_N/EtOAc/hexanes (1:1:18) solvent system to afford a 13-*cis*/*trans* mixture of the protected coupling product. This mixture was further separated by HPLC under straight-phase conditions on a Luna 5 µm Silica (2) 100 Å, 250 × 10 mm column (Phenomenex) using hexanes/triethylamine (99:1) to furnish pure title compound **1** (15 mg, 51%). The 13*E*/*Z* isomeric ratio was found to be 1:9 as judged by HPLC analysis. The UV spectrum (hexanes/triethylamine, 99:1) of the title compound showing a triplet band of absorption at λ_max_ 294, 308, 322 nm was in agreement with the reported values by Rodriguez and Spur [14]. For increased stability, compound 1 was stored in its silyl ether and ester protected form and was subjected to deprotection upon need for biological studies with live cells and recombinant enzymes, which further confirmed its exact stereochemical structure [13].

### 3.9. Conversion of Compound ***1*** to RCTR1, RCTR1 and RCTR3

The stereochemical configuration of the epoxy group and double-bond geometry in compound **1** was confirmed by converting analytical amounts (~3 ng, each) of **1** to RCTR1, RCTR2, and RCTR3, naturally occurring and bioactive cysteinyl-containing products biosynthesized from docosahexaenoic acid. This was achieved by opening of the epoxide with reduced glutathione, l-cysteinylglycine, and l-cysteine, respectively, in MeOH/H_2_O/Et_3_N, followed by desilylation and ester hydrolysis under *^n^*Bu_4_NF/AcOH and LiOH conditions. Synthesis of RCTR1, RCTR2, and RCTR3 was confirmed by a liquid chromatography and tandem mass spectrometry (LC-MS/MS)-based approach. Chromatographic retention times and fragmentation spectra of each RCTR compound were matched to those of the reference and commercially available standard.

## 4. Conclusions

In summary, we have developed an efficient strategy towards the synthesis of the TES ether and methyl esterderivative of 7,8(*S*,*S*)-oxido-17(*S*)-hydroxy-4(*Z*),9(*E*),11(*E*),13(*Z*),15(*E*),19(*Z*)-docosahexaenoic acid (**1**). The reported method is convergent and highly stereocontrolled, and employs simple, commercially cheap starting materials. Key features included the Sharpless epoxidation to forge the oxirane ring, and a *cis*-selective Wittig coupling to construct the critical 13*Z*-olefinic geometry. This synthetic route provides an alternative approach to access this labile, yet indispensable natural product. In its deprotected state, this compound was pivotal in elucidating its role and involvement in the biosynthesis of the naturally occurring, protective, and anti-inflammatory resolvin D1, resolvin D2, and RCTR1 [13]. These efforts provide invaluable insights and the molecular basis for the biogenesis of these potent products and highlight the central role of this epoxy polyunsaturated fatty acid. Continued research exploring the mechanisms of action and formation of these autacoids may contribute to the development of new therapeutic agents.

## Data Availability

The original contributions presented in this study are included in the article/Supplementary Materials. Further inquiries can be directed to the corresponding author.

## Supplementary Materials

The following supporting information can be downloaded at: https://www.mdpi.com/article/10.3390/molecules30081858/s1, Figures S1–S14. Spectroscopic data (^1^H NMR, ^13^C NMR, HPLC, UV, LC-MS/MS) of compounds **1**, **2**, **3**, **4**, **5**, **6**, **8**, **9**, **RCTR1**, **RCTR2**, **RCTR3**, as well as select intermediate precursors.
