# Supplementary material for: On the Total Synthesis of 7,8(S,S)-Epoxy-17(S)-hydroxy-4(Z),9(E),11(E),13(Z),15(E),19(Z)-docosahexaenoic Acid Derivative
Source: Molecules. 2025 Apr 21;30(8):1858. doi: 10.3390/molecules30081858 (PMC12029207; doi:10.3390/molecules30081858)
Supplement: Supplementary file 1 [file molecules-30-01858-s001.zip › molecules-3543805-supplementary.pdf]

## Supplementary Materials:

# On the total synthesis of 7,8(*S*,*S*)-epoxy-17(*S*)-hydroxy-4(*Z*),9(*E*),11(*E*),13(*Z*),15(*E*),19(*Z*)-docosahexaenoic acid derivative

Robert Nshimiyimana,[1,2,*] Charles N. Serhan,[1] and Nicos A. Petasis[2]

[1]Center for Experimental Therapeutics and Reperfusion Injury, Department of Anesthesiology, Perioperative and Pain Medicine, Brigham and Women's Hospital and Harvard Medical School, Boston, MA 02115, USA

[2]Department of Chemistry and Loker Hydrocarbon Research Institute, University of Southern California, Los Angeles, CA 90089, USA

*E-mail: rnshimiyimana@bwh.harvard.edu

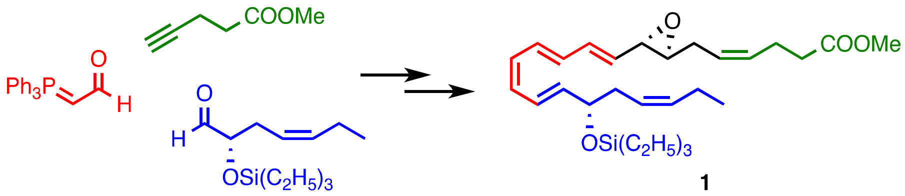


**1**

## Table of Contents

| | | |
|---|---|---|
| S2 | ................ | General Information |
| S5 | ................ | $^{1}$H and $^{13}$C NMR spectra of compound **4** and its precursor |
| S7 | ................ | $^{1}$H and $^{13}$C NMR spectra of precursor to compound **5** |
| S8 | ................ | $^{1}$H and $^{13}$C NMR spectra of compound **6** and its precursor |
| S10 | ................ | $^{1}$H and $^{13}$C NMR spectra of compound **2** |
| S11 | ................ | $^{1}$H and $^{13}$C NMR spectra of compound **8** |
| S12 | ................ | $^{1}$H and $^{13}$C NMR spectra of compound **9** |
| S13 | ................ | $^{1}$H and $^{13}$C NMR spectra of compound **3** |
| S15 | ................ | HPLC chromatograms of compound **1** |
| S16 | ................ | UV spectrum of compound **1** |
| S17 | ................ | LC-MS/MS of RCTR1 |
| S18 | ................ | LC-MS/MS of RCTR2 |
| S19 | ................ | LC-MS/MS of RCTR3 |

**General Information**

Unless stated otherwise, all reactions were carried out in a flame-dried round-bottom flask equipped with a stirring bar under argon atmosphere. Reactions were carried out using DriSolv solvents purchased from VWR (Radnor, PA, USA). Research-grade reagents were purchased and used without further purification from suppliers including Sigma-Aldrich (St. Louis, MO, USA), Strem Chemicals (Newburyport, MA, USA), Combi-Blocks (San Diego, CA, USA), TCI America (Portland, OR, USA), and Alfa Aesar (Ward Hill, MA, USA).

Reaction progress was monitored using EMD analytical thin-layer chromatography (TLC) plates, Silica Gel 60 F254 (Sigma-Aldrich, St. Louis, MO, USA). TLC plates were visualized through UV absorbance, or staining techniques including vanillin, phosphomolybdic acid, potassium-permanganate, or ninhydrin followed by heating. Purification was carried out by manual flash chromatography or by automated system using Biotage Isolera One (Biotage, Uppsala, Sweden) on Silica Gel (100-200 mesh). The final product was further purified by HPLC on Agilent 1100 Series or 1260 Infinity HPLC system (Agilent, Santa Clara, CA, USA) equipped with a binary pump and diode array detector on a Luna 5 µm Silica (2) 100 Å, 250 × 10 mm column (Phenomenex, Torrance, CA, USA) under straight-phase conditions.

NMR data were procured on Varian NMR spectrometers (Varian Inc, Palo Alto, CA, USA) at 400, 500 or 600 MHz, respectively, for $^{1}$H NMR, and at 101, 126 or 151 MHz, for $^{13}$C NMR. $^{1}$H and $^{13}$C chemical shifts (δ) are recorded in parts per million (ppm), relative to the residual protium and carbon solvent resonances in $^{1}$H NMR (*e.g.*, Chloroform-*d*, $^{1}$H = 7.26 ppm; benzene-$d_6$, $^{1}$H = 7.16 ppm; and methanol-$d_4$, $^{1}$H = 3.31 ppm) and $^{1}$C NMR (*e.g.*, Chloroform-*d*, $^{13}$C = 77.16 ppm; benzene-$d_6$, $^{13}$C = 128.06 ppm; and methanol-$d_4$, $^{13}$C = 49.00 ppm). Splitting patterns are denoted by s, d, t, dd, td, ddd, and m and refer to the respective multiplicities; singlet, doublet, triplet, doublet of doublets, triplet of doublets, doublet of doublet of doublet and multiplet.

Liquid chromatography and tandem mass spectrometry (LC-MS/MS) data were procured on a Sciex Triple Quad 7500 (Sciex, Framingham, MA, USA) coupled with an

ExionLC analytical HPLC (Sciex, Framingham, MA, USA) on a Kinetex 2.6 μm PS C18 100 Å, 100 × 3.0 mm column (Phenomenex, Torrance, CA, USA) under reversed phase conditions.

**Selected $^{1}H$ and $^{13}C$ NMR Spectra**

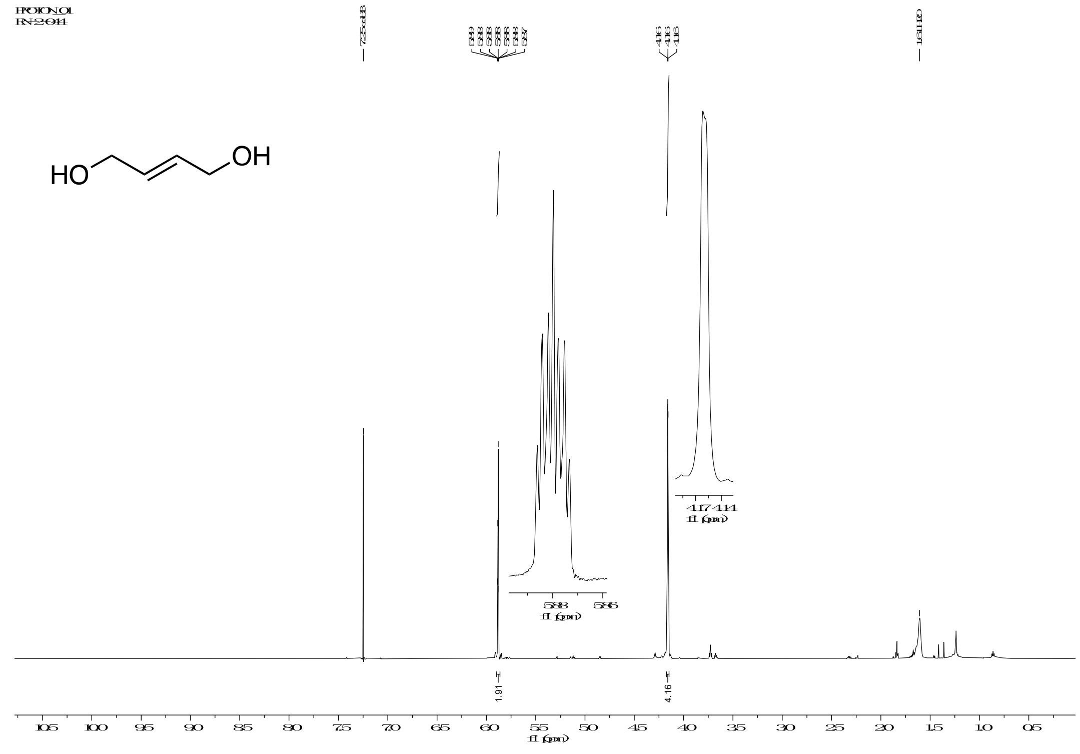


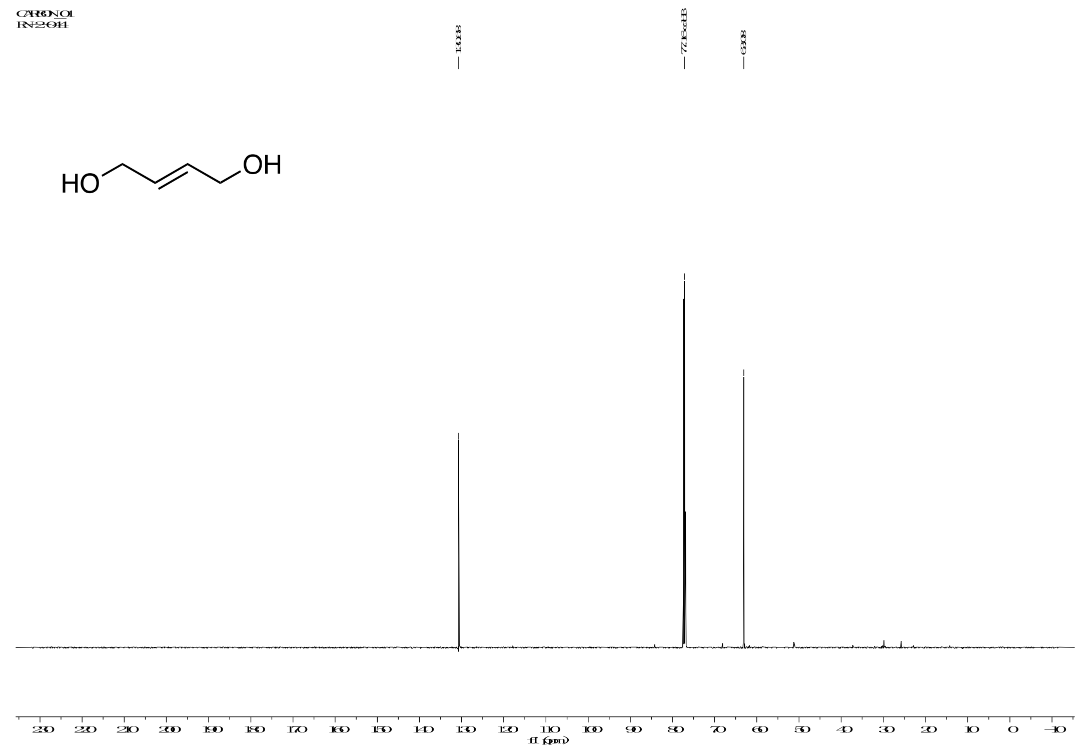


**Figure S1**. $^{1}$H and $^{13}$C NMR spectra of *trans*-2-butene-1,4-diol precursor to **4**.

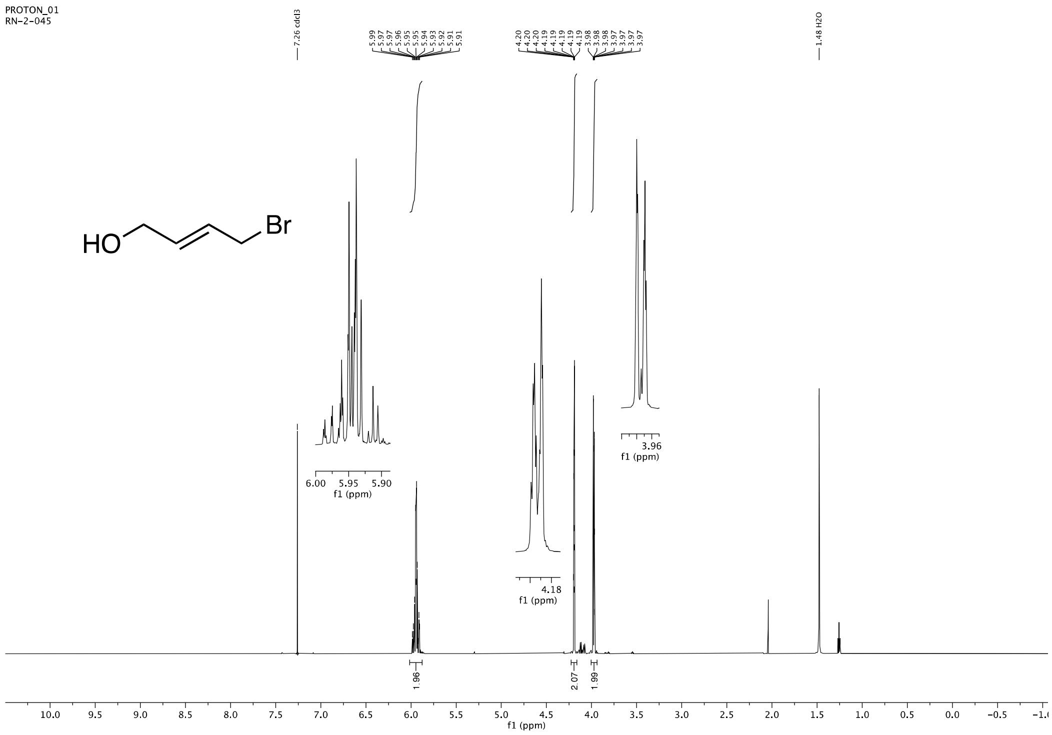

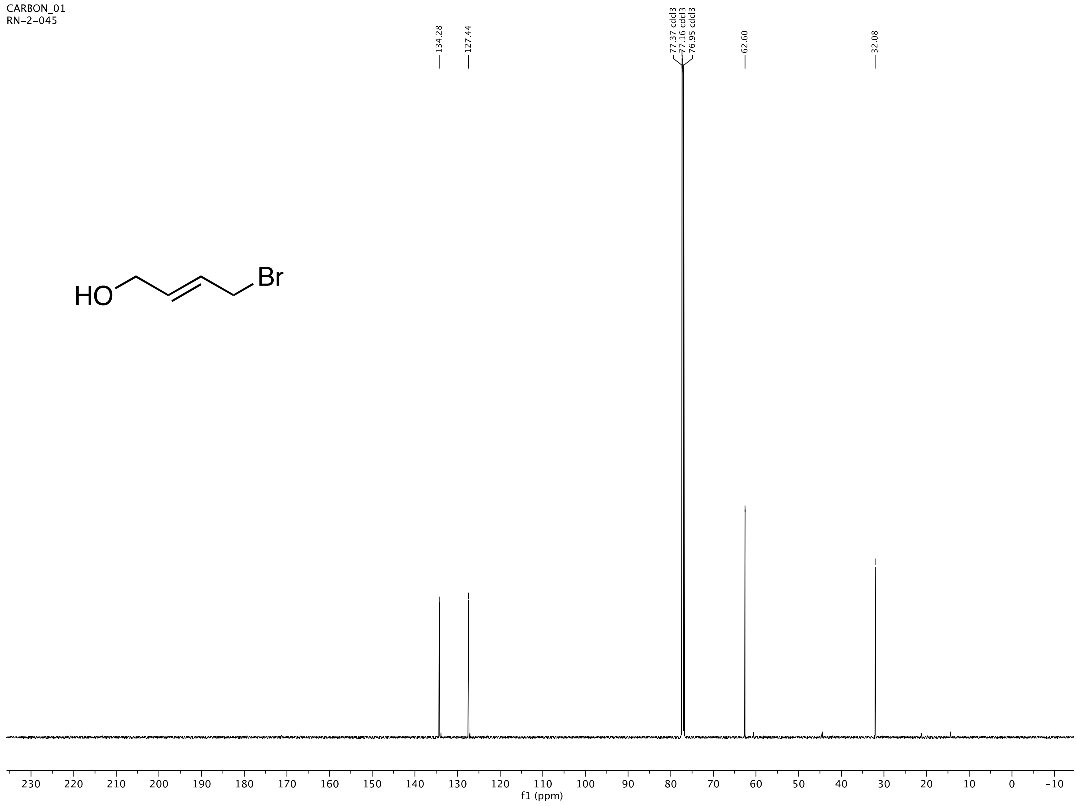

**Figure S2**. $^{1}$H and $^{13}$C NMR spectra of compound **4**.

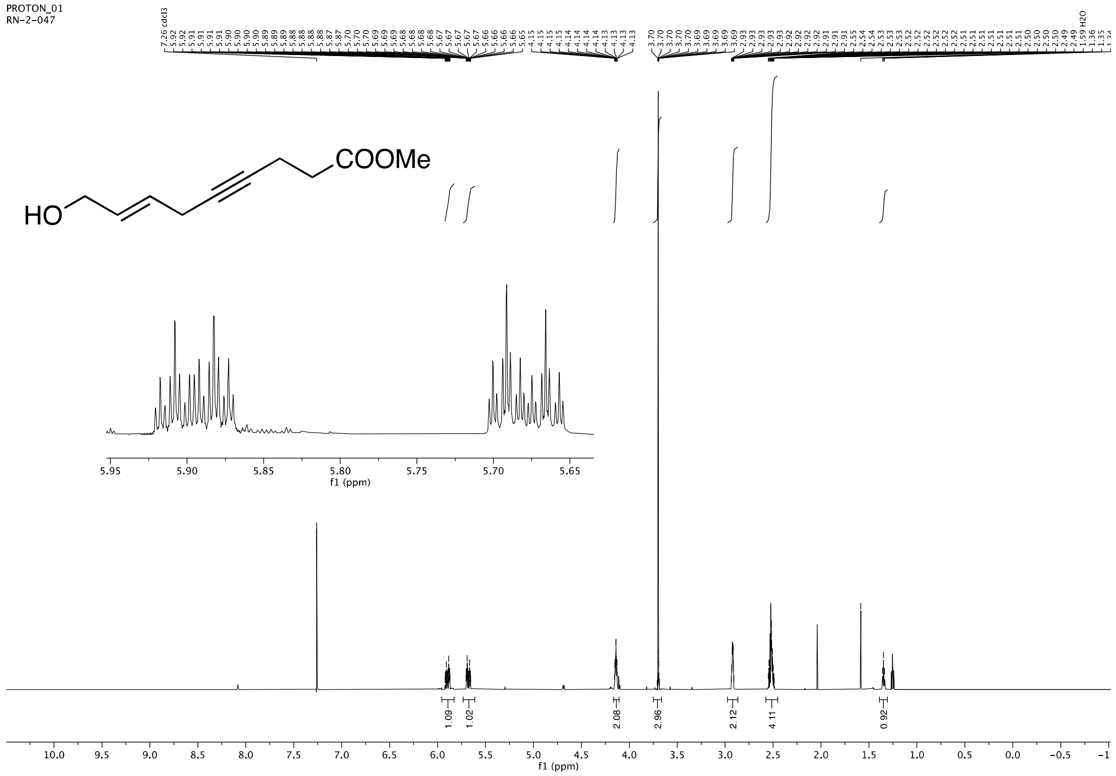


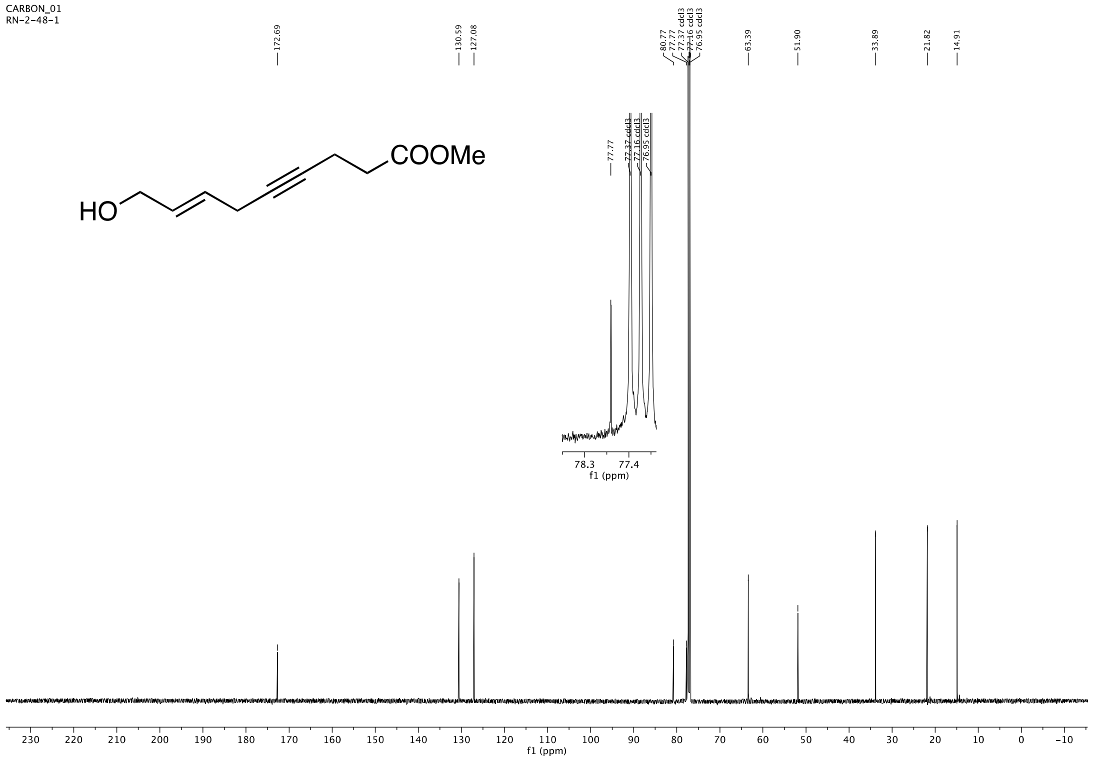


**Figure S3**. $^{1}$H and $^{13}$C NMR spectra of skipped enyne precursor to **5**.

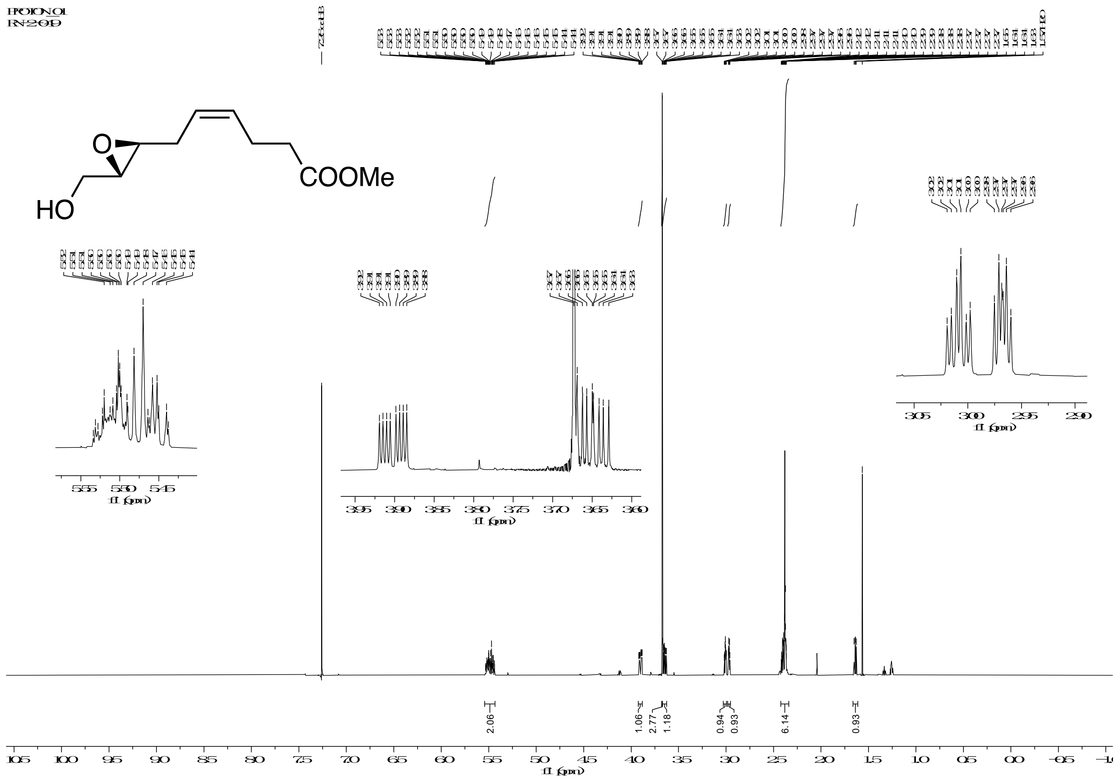

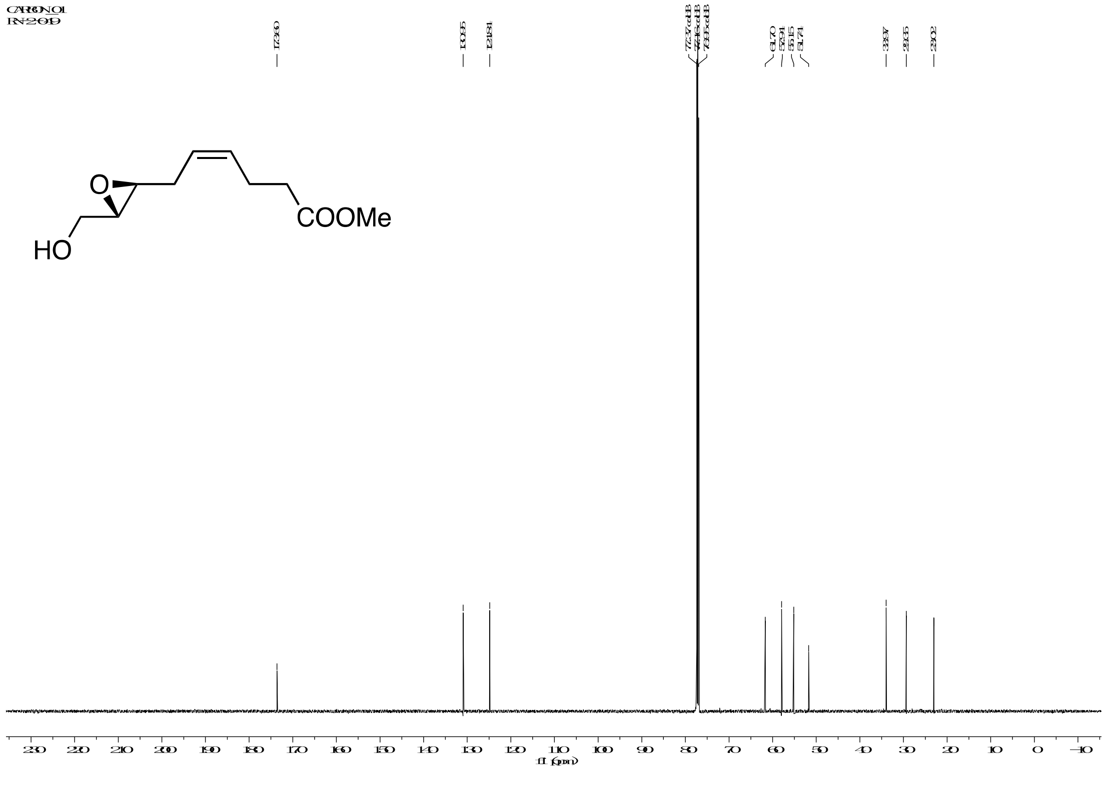

**Figure S4**. $^{1}H$ and $^{13}C$ NMR spectra of epoxyalcohol precursor to **6**.

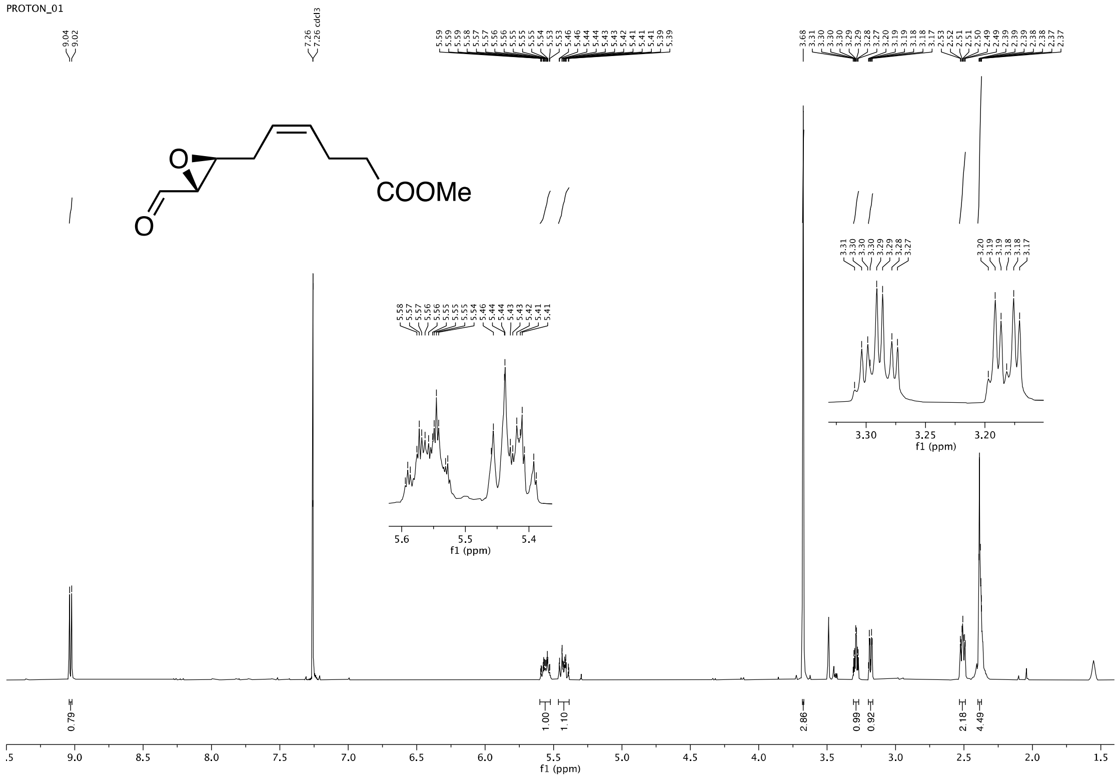

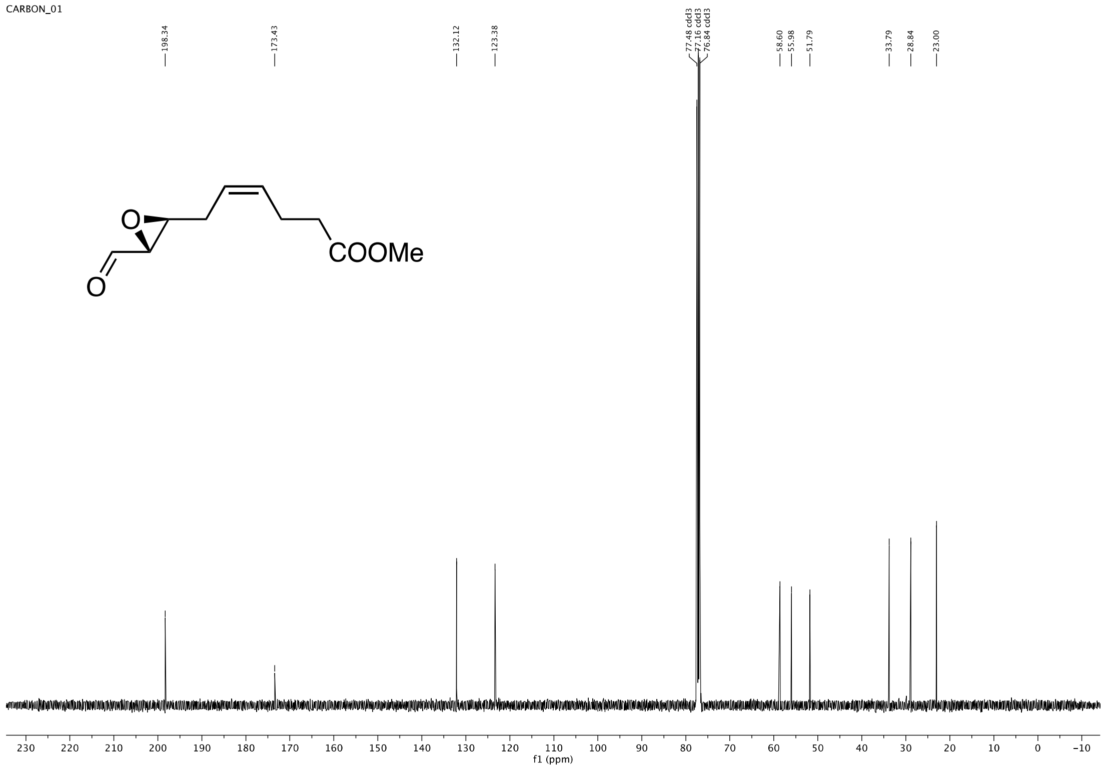

**Figure S5**. $^{1}H$ and $^{13}C$ NMR spectra of epoxyaldehyde **6**.

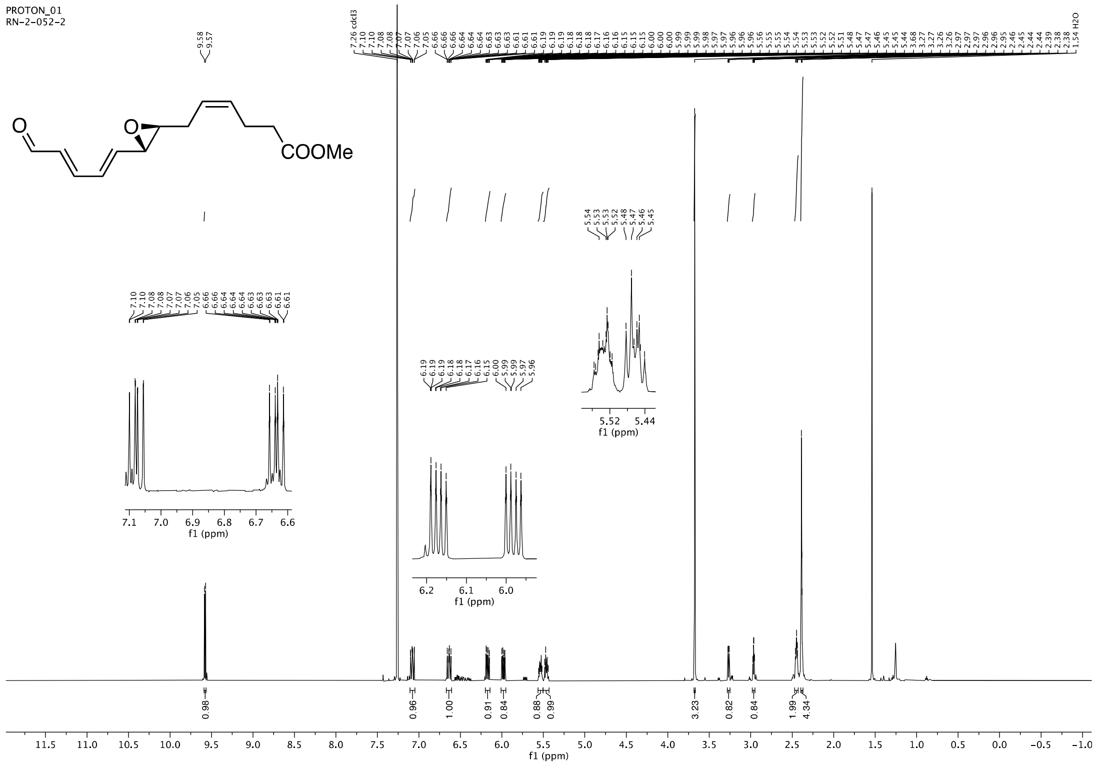


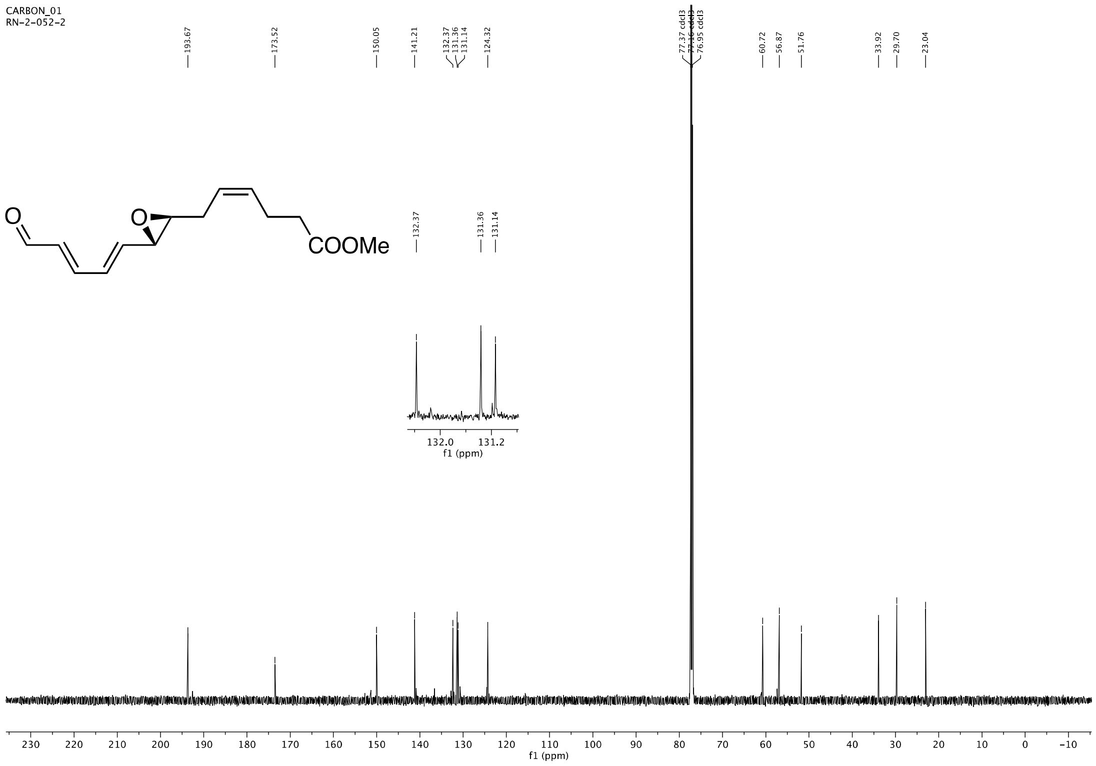


**Figure S6**. $^{1}H$ and $^{13}C$ NMR spectra of $C_1$–$C_{13}$ epoxydienal **2**.

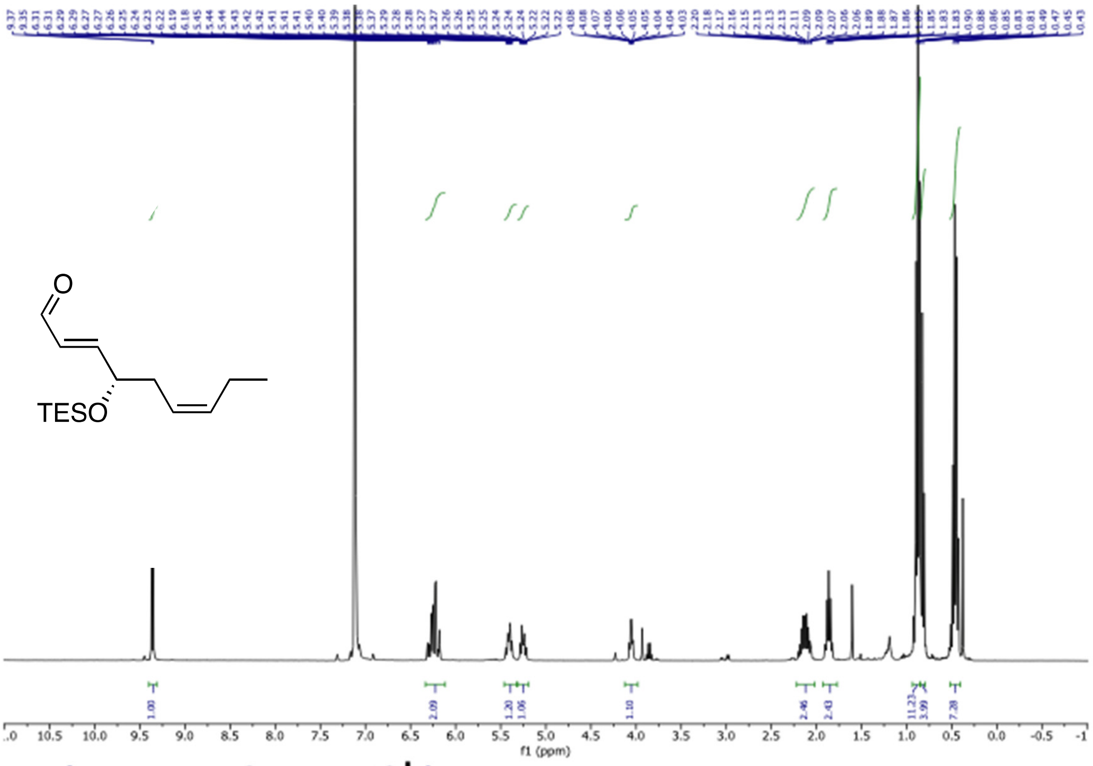

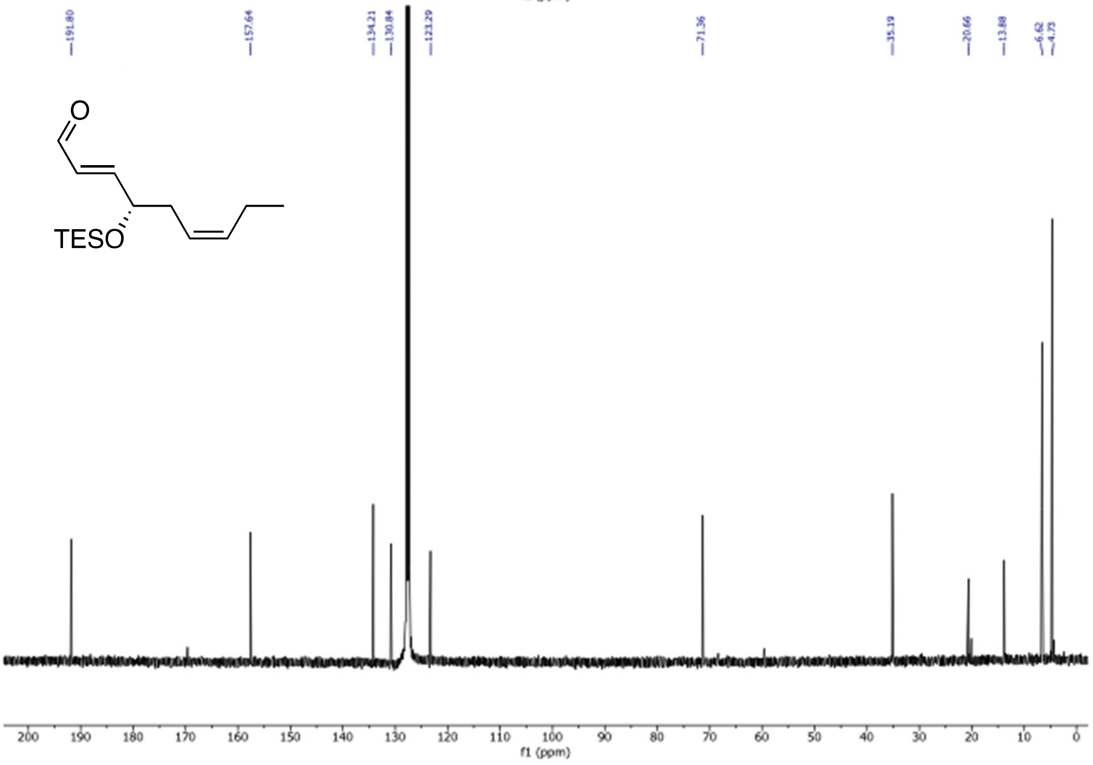

**Figure S7**. $^{1}H$ and $^{13}C$ NMR spectra of compound **8**.

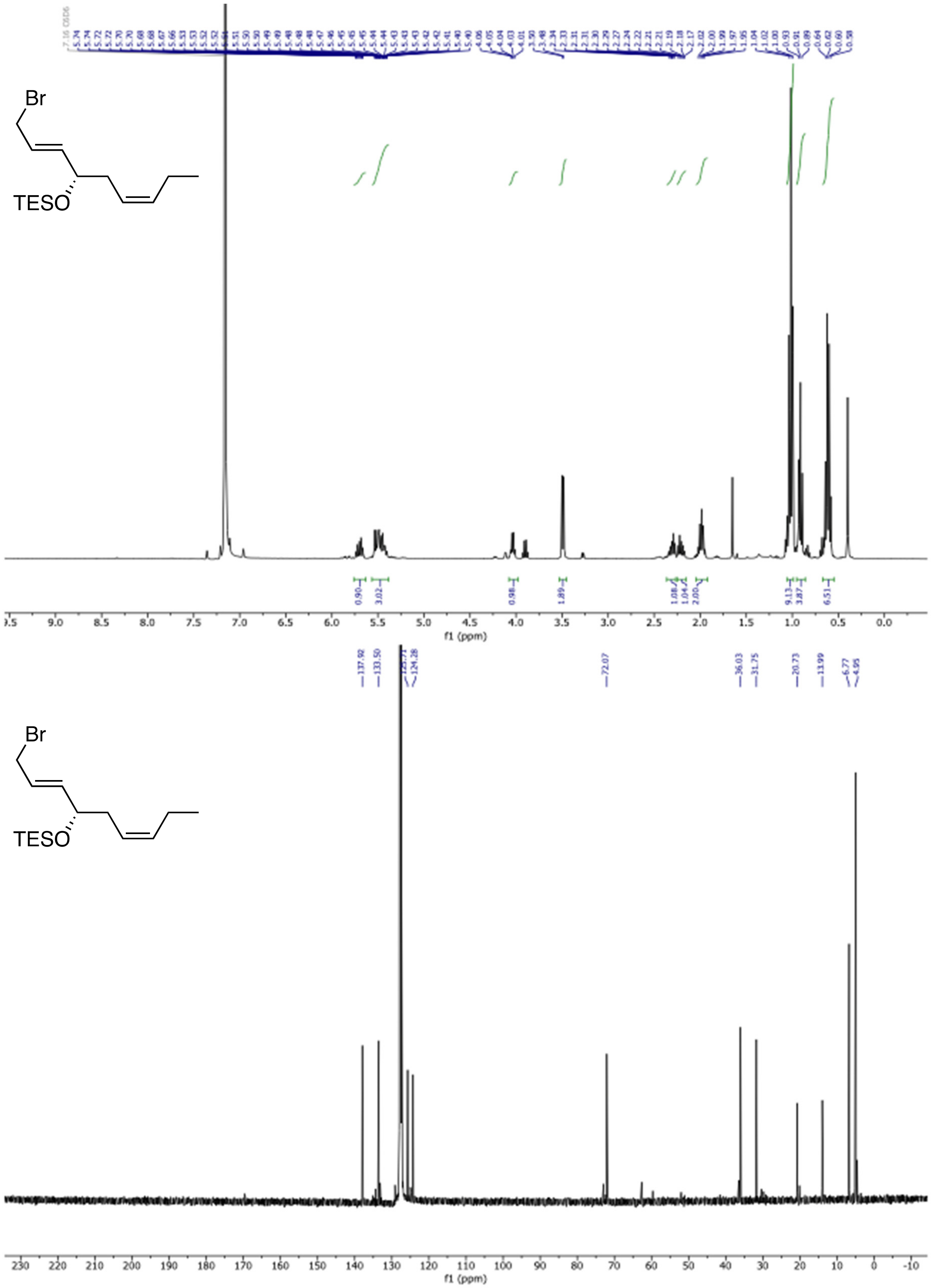


**Figure S8**. ¹H and ¹³C NMR spectra of allylic bromide **9**.

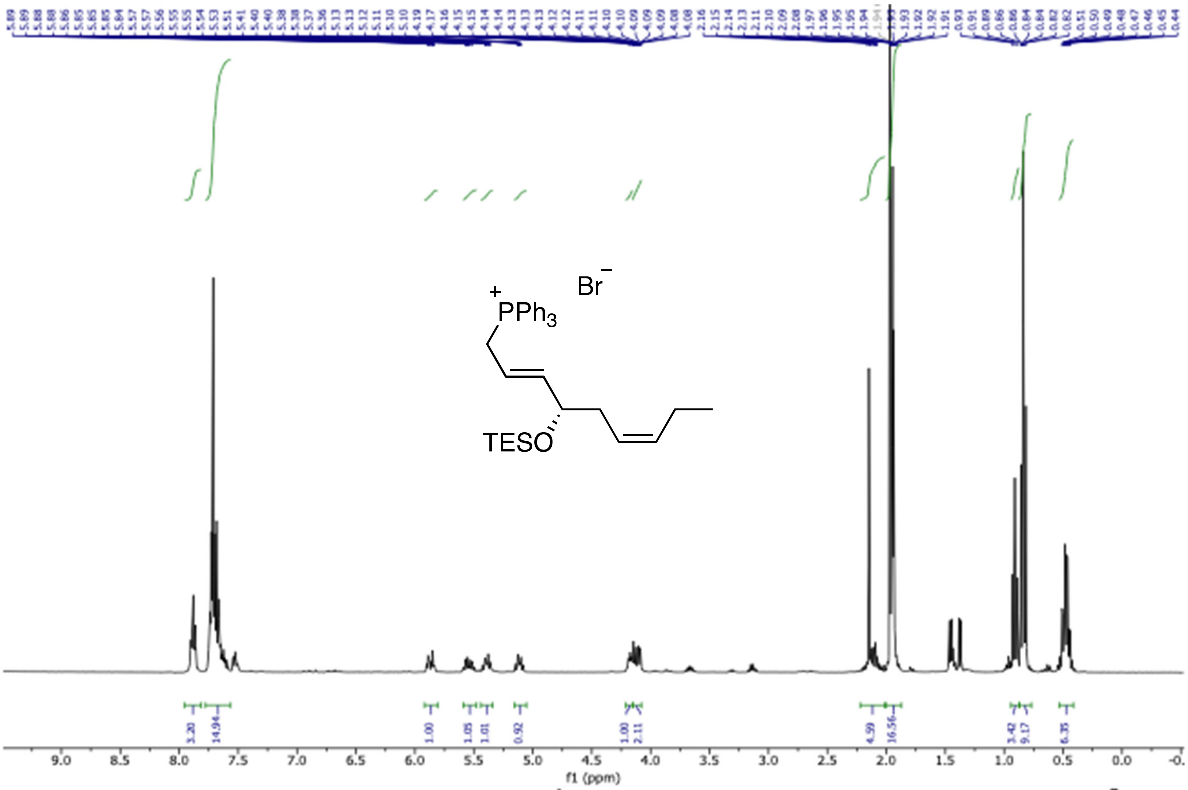

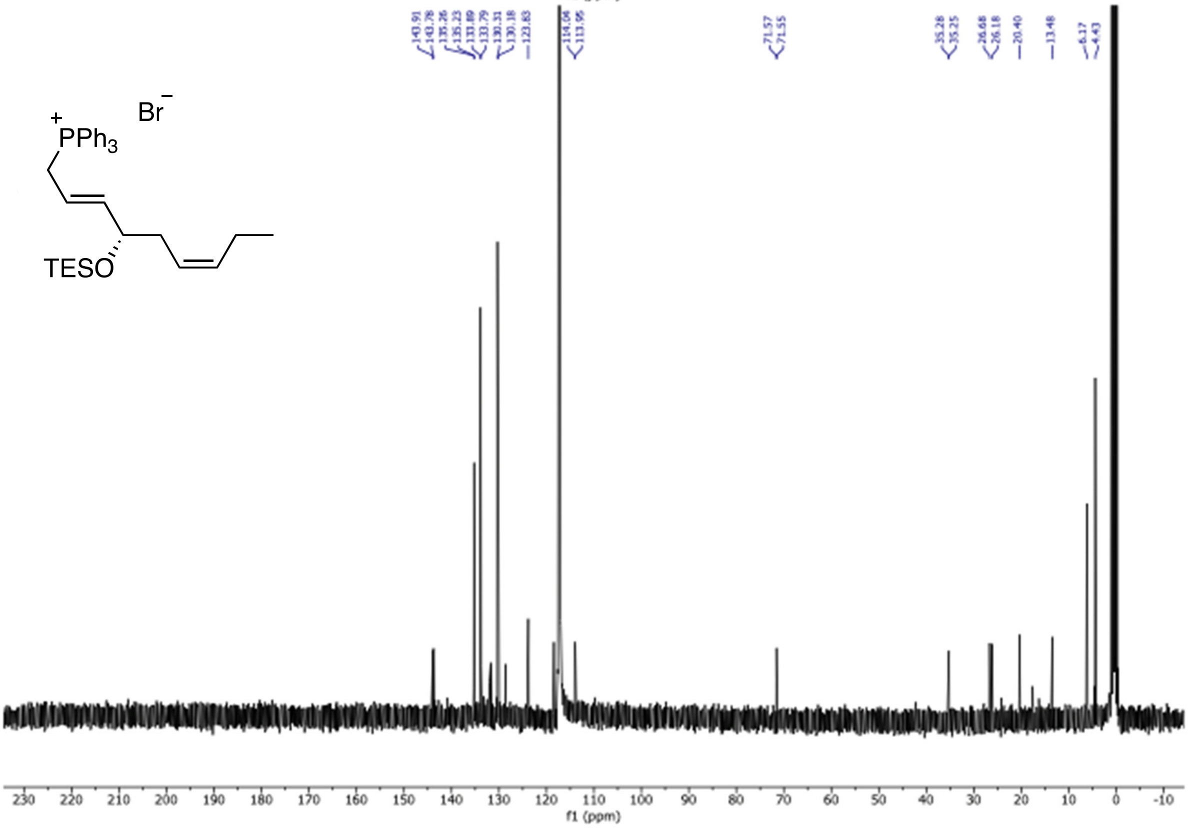

**Figure S9.**$^{1}$H and $^{13}$C NMR spectra of phosphonium bromide **3**.

## UV, HPLC, and LC-MS/MS Chromatograms and Spectra

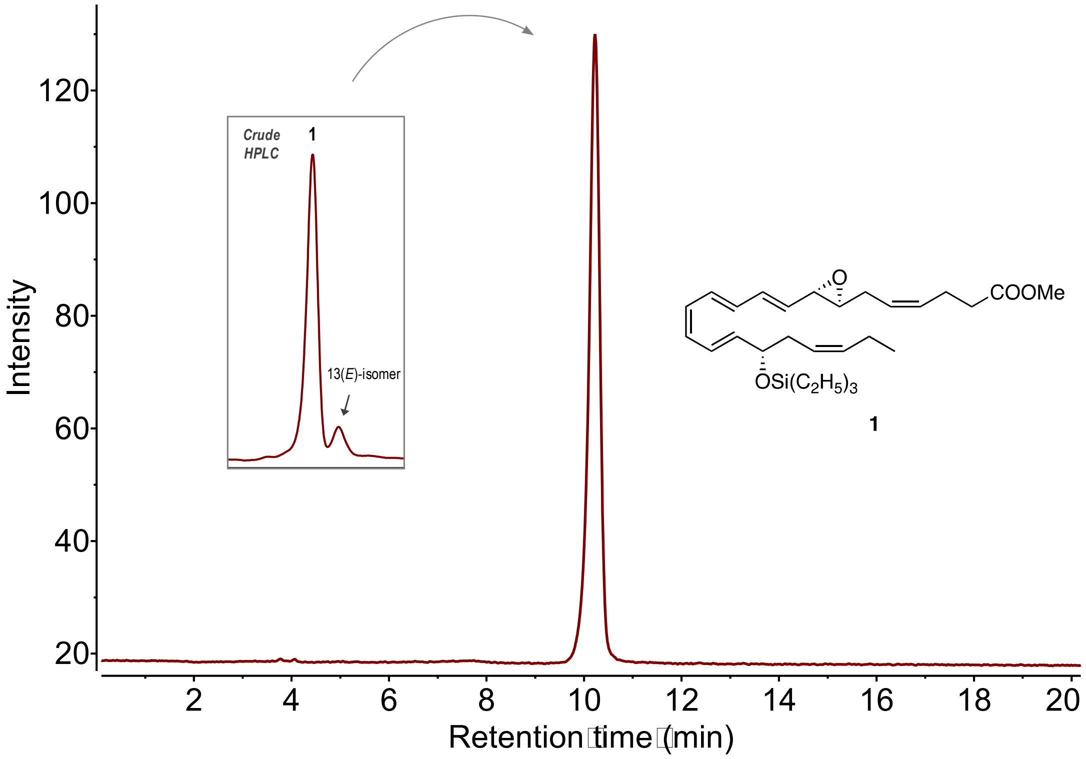


**Figure S10**. HPLC chromatogram of purified target compound **1**. *Inset*, chromatographic trace of crude mixture showing the presence of the 13(*E*)-geometric isomer (~1:9 *E*/*Z* ratio).

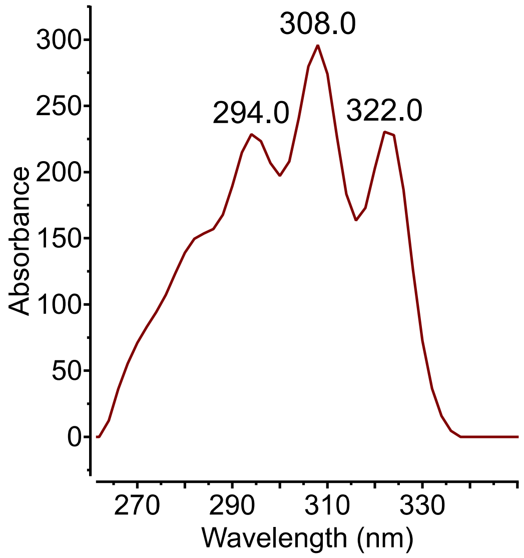


**Figure S11**. UV absorbance spectrum of compound **1**.

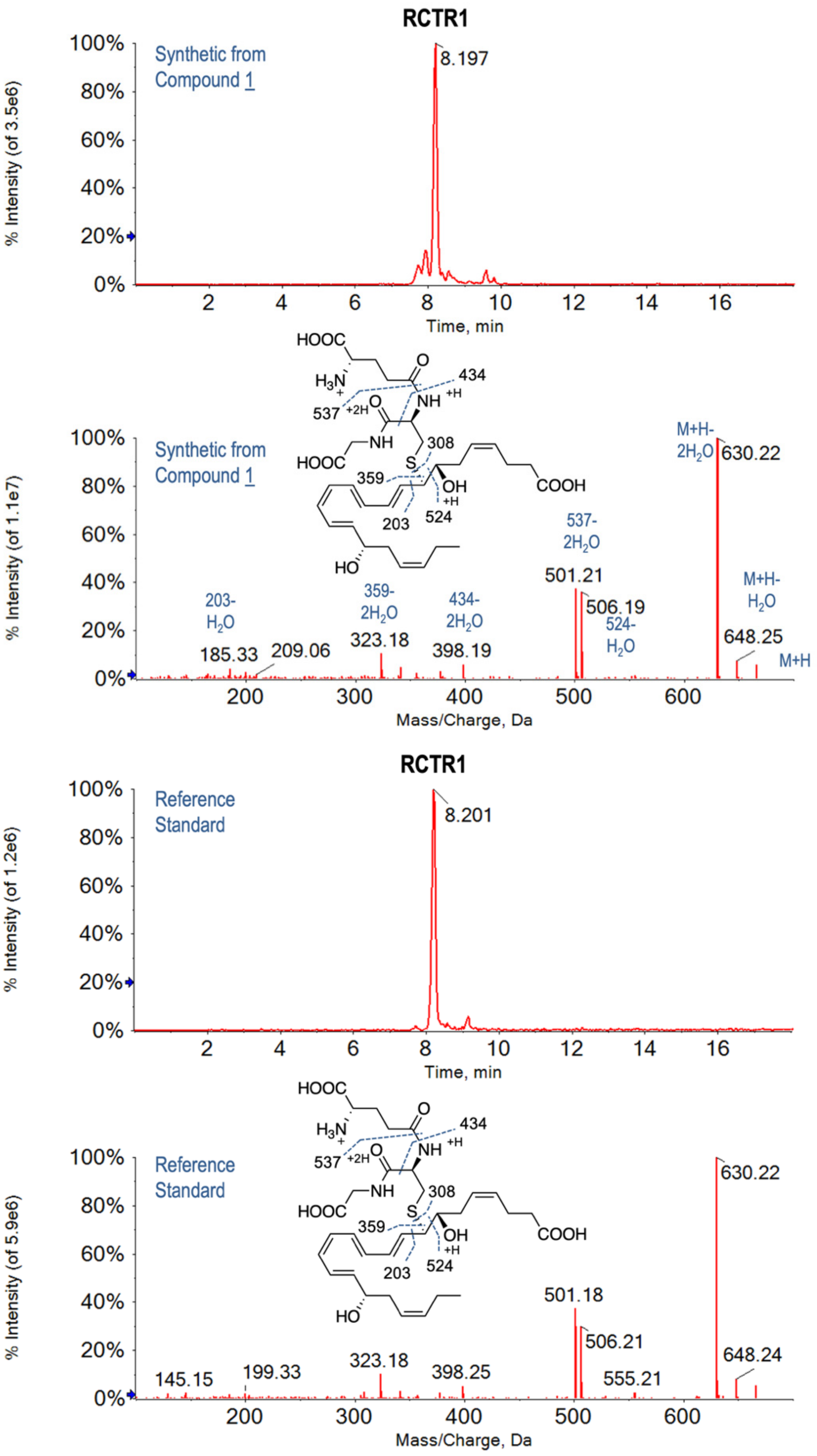


**Figure S12**. Retention time and $MS^2$ fragmentation spectrum of RCTR1, synthesized from compound **1**, and matching of these physical properties to the reference standard.

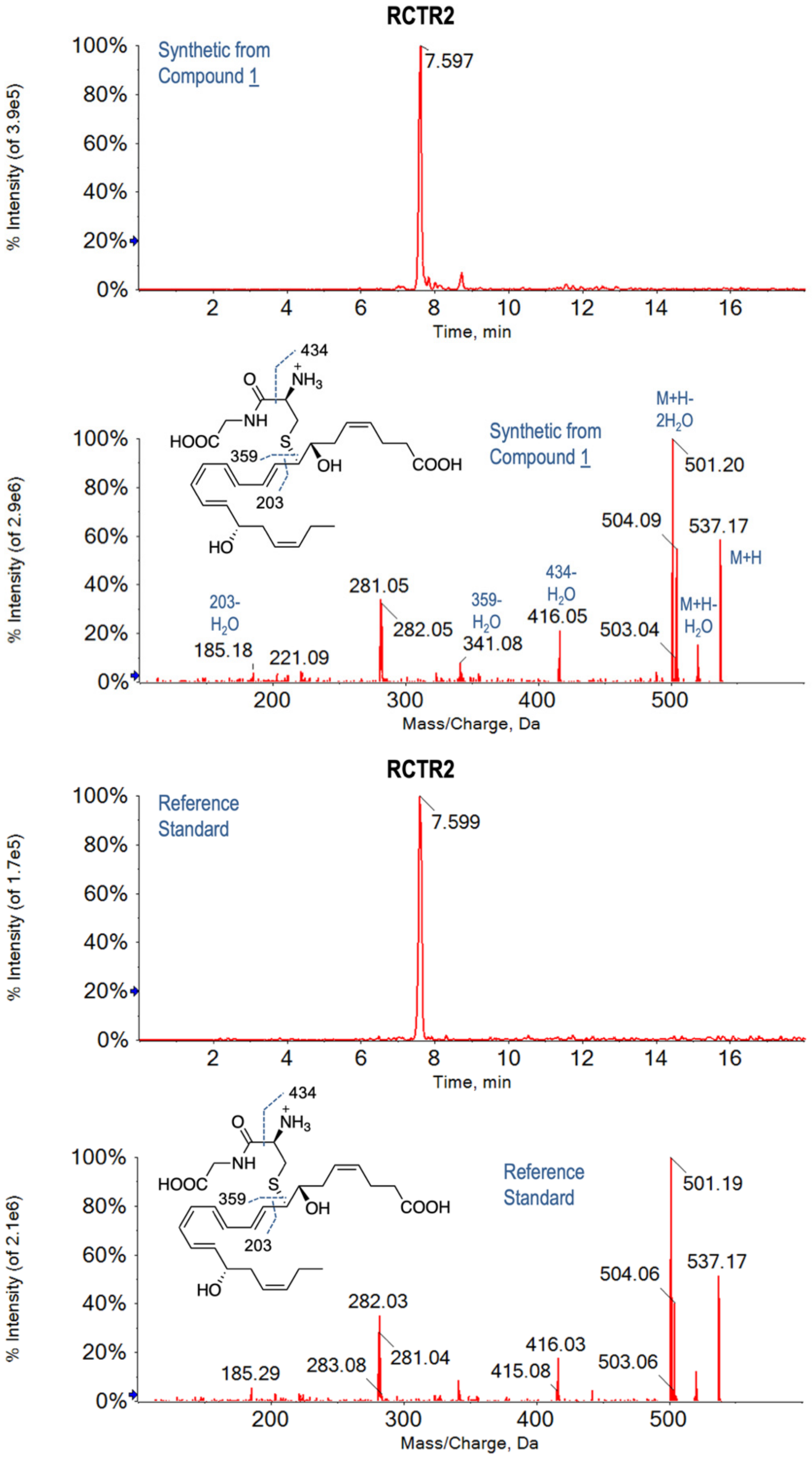


**Figure S13**. Retention time and MS² fragmentation spectrum of RCTR2, synthesized from compound **1**, and matching of these physical properties to the reference standard.

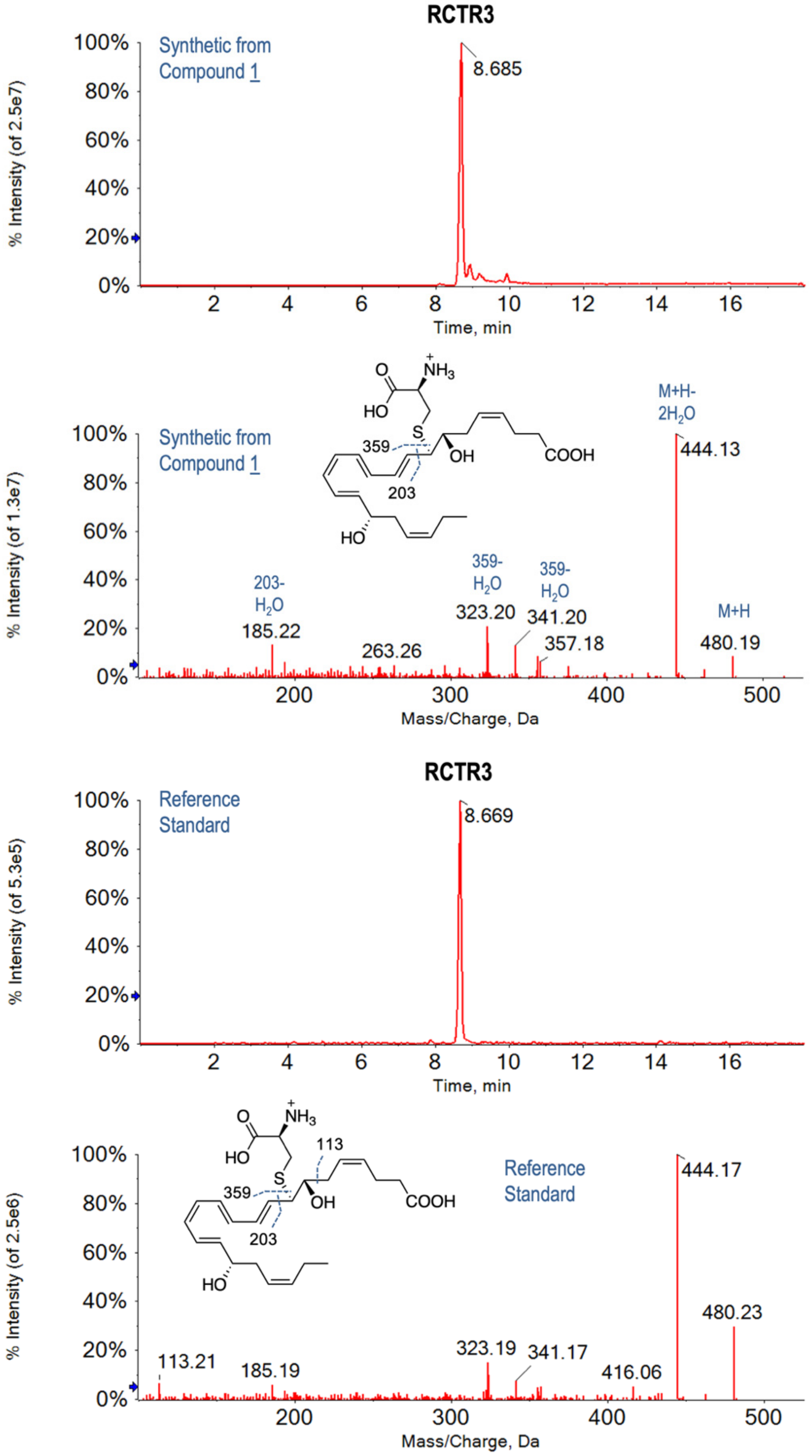


**Figure S14**. Retention time and MS² fragmentation spectrum of RCTR3, synthesized from compound **1**, and matching of these physical properties to the reference standard.
